# Identification and functional characterization of the MYB transcription factor *GmMYBLJ* in soybean leaf senescence

**DOI:** 10.3389/fpls.2025.1533592

**Published:** 2025-01-24

**Authors:** Guohua Bao, Xiao Xu, Jing Yang, Juanjuan Liu, Tianran Shi, Xiaoxuan Zhao, Xuyan Li, Shaomin Bian

**Affiliations:** ^1^ College of Plant Science, Jilin University, Changchun, Jilin, China; ^2^ Institute of Agricultural Biotechnology, Jilin Academy of Agricultural Sciences (Northeast Agricultural Research Center of China), Changchun, Jilin, China

**Keywords:** soybean, *GmMYBLJ*, leaf senescence, *Arabidopsis*, dark, stress

## Abstract

Leaf senescence is an important agronomic trait that significantly influences the quality and yield of soybeans. v-Myb avian myeloblastosis viral oncogene homolog (MYB) transcription factors are considered crucial regulators governing leaf senescence, which can be utilized to improve agronomic traits in crops. However, our knowledge regarding the functional roles of soybean MYBs in leaf senescence is extremely limited. In this study, *GmMYBLJ*, a *CCA1-like MYB*, was identified and functionally characterized with respect to leaf senescence. The GmMYBLJ protein is localized in the nucleus, and a high accumulation of its transcripts was observed in nodules and embryos. Notably, *GmMYBLJ* was highly expressed in soybean senescent leaves and was transcriptionally induced by dark or NaCl treatment, as confirmed by histochemical GUS staining analysis. Ectopic overexpression of *GmMYBLJ* in *Arabidopsis* not only led to earlier leaf senescence, reduced chlorophyll content, and increased MDA accumulation but also promoted the expression of several *WRKY* family transcription factors and senescence-associated genes, such as *SAG12* and *ORE1*. Further investigation showed that overexpression of *GmMYBLJ* accelerated *Arabidopsis* leaf senescence under darkness and in response to *Pst* DC3000 infection. Moreover, transgenic soybean plants overexpressing *GmMYBLJ* grew faster and exhibited accelerated senescence under salt stress. DAB staining analysis showed that *GmMYBLJ* induced ROS accumulation in soybean hairy roots and *Arabidopsis* leaves. Collectively, our results provided useful information into the functional roles of *GmMYBLJ* in both age-dependent and stress-induced senescence.

## 1 Introduction

Leaf senescence is the final phase of leaf development, characterized by chloroplast degeneration and macromolecular catabolism (Lei et al., 2023). It is often accompanied by the accumulation of reactive oxygen species (ROS). Senescence is a natural process associated with aging, which can facilitates the release and recycling of nutrients for use by other developing organs or storage for the next generation or season (Zhou and Yang, 2023). However, adverse environmental factors, such as darkness, shade, nutrient deficiency, low temperature, and both abiotic and biotic stresses, often lead to premature senescence, thereby reducing the yield and quality of crop plants (Antonietta et al., 2024). Increasing evidence suggests that the process of leaf senescence is highly coordinated and exquisitely regulated by both internal and external stimuli (Sasi et al., 2022; Zhao et al., 2022; Cao et al., 2023). Thus, studying the regulators of leaf senescence and their regulatory mechanisms is agronomically important for manipulating leaf traits in crop plants.

v-Myb avian myeloblastosis viral oncogene homolog (MYB) proteins constitute a large family of transcription factors found in all eukaryotes, characterized by a varying number of MYB domains that contribute to their DNA-binding capacity. Based on the number of MYB domains, the MYB family can be classified into four categories: 1R, 2R, 3R, and 4R MYB (Biswas et al., 2023). It is well known that *MYBs* are involved in the regulation of many plant developmental and growth processes, as well as responses to abiotic and biotic stresses (Thakur and Vasudev, 2022; Biswas et al., 2023). Notably, several studies have shown that *MYBs* play positive roles in leaf senescence through hormonal pathways. For example, overexpression of MYB HYPOCOTYL ELONGATION-RELATED (*AtMYBH*) (a 1R-MYB gene) in *Arabidopsis* accelerated ethylene- and Abscisic Acid (ABA)-induced leaf senescence, while *AtMYBH* negatively regulated auxin homeostasis by inhibiting the expression of *AtDFL1/AtGH3.6* and *AtDFL2/AtGH3.10* (Huang et al., 2015). Similarly, *AtMYBL* (a R-R-type MYB-like gene) promoted leaf senescence in *Arabidopsis*, and its expression could be induced by the phytohormone ABA (Zhang et al., 2011). Likewise, overexpression of *NtMYB1* in Narcissus or *Arabidopsis* promoted ABA production and decreased the level of gibberellin A1, leading to leaf senescence symptoms such as leaf yellowing and a decrease in photosystem II fluorescence (Yang et al., 2023). In addition, *MYBs* are positively involved in the regulation of dark-induced leaf senescence. A good example is that mutation of *Arabidopsis GLABRA* (*AtGL1*), which resulted in reduced susceptibility to dark-induced aging (Eckstein et al., 2019), implying its functional role in governing the initiation and progression of leaf senescence. However, a few reports have shown that some *MYBs* might negatively regulate leaf senescence. For example, *AtMYBR1/AtMYB44* and its homolog *OsMYB102* function as negative regulators of leaf senescence by affecting the ABA pathway (Jaradat et al., 2013; Piao et al., 2019). Further investigation revealed that AtMYB44 directly bound to the promoter of *AtBBX14* and activated its expression, which subsequently functioned as a negative regulator of N starvation-induced senescence through *AtEIN3* (Buelbuel et al., 2023). Evidently, different *MYB* members might exhibit functionally diverse roles in the regulation of leaf senescence. To date, several *MYBs* involved in the regulation of leaf senescence have been identified in various plant species, such as rice (Akhter et al., 2019; Piao et al., 2019), cottonwood (Lu et al., 2020), sunflower (Moschen et al., 2016), and *Jatropha curcas* (Chen et al., 2022). However, leaf senescence-associated *MYBs* in additional plant species need to be identified to meet the requirements of molecular breeding design.

Soybean (*Glycine max*) is an economically important crop due to its storage of proteins and oils. Leaf senescence is a crucial trait that significantly impacts the quality and yield of crops. Generally, delaying leaf senescence is considered to promote higher yield and quality (Antonietta et al., 2024). However, early soybean harvesting is one of the most desirable agricultural traits in high-latitude regions (Zhu et al., 2023; Gao et al., 2024), and early leaf senescence might contribute to shortening the maturation period. To date, a number of regulators have been identified that control leaf senescence in soybean, such as the *GmCRY1s-GmDELLAs-GmWRKY100* regulatory cascade (Li et al., 2024), *GmNACs* (*NAC81*, *NAC30*, *NAC65*)-*SAG*s (Ferreira et al., 2020; Fraga et al., 2021), *GmELS1* (Yamatani et al., 2021), *GmBiP* (Carvalho et al., 2014), *GmSARK* (Xu et al., 2011), *GmWRKY53b*, and *GmCIB1* (Meng et al., 2013). Recently, it was reported that *GmMYB128* might be a positive regulator of nodule senescence in soybean (Liu et al., 2024). However, little is known about the functional roles of *MYBs* in soybean leaf senescence.

In the current study, our primary objective is to characterize a soybean CCA1-like MYB, GmMYBLJ, and explore its functional roles in governing leaf senescence and stress response. Our results suggest that GmMYBLJ might act as a positive regulator in age-dependent and stress-induced leaf senescence by regulating WRKY and senescence-associated genes. These findings expand the understanding of the regulatory functions of MYB transcription factors in soybean senescence, which could inform breeding strategies aimed at optimizing leaf senescence and stress tolerance.

## 2 Materials and methods

### 2.1 Plant materials and growth conditions

Seedlings of *Arabidopsis* (Columbia-0), tobacco (*Nicotiana benthamiana*), and soybean (*Glycine max*, cv “Jilin 32”) were grown under 16 h/8 h (light/dark) photoperiod cycle at 25°C with 70%–80% relative humidity. These conditions were used for the treatments and sample collection in the study. Compound leaves were collected every 15 days (15, 30, 45, 60, and 75 days) once the fourth triple compound leaf emerged from the seedling (V4 stage). Soybean plants were grown at the experimental station of Jilin University (Changchun, China). Soybean tissues, including root, stem, leaf, flower, and nodule, were collected randomly during flowering, as well as embryos at six different developmental stages (10, 20, 30, 40, 50, and 60 days after pollination [DAP]).

### 2.2 Generation of transgenic *Arabidopsis* and soybean lines as well as transgenic hairy roots

To generate transgenic *Arabidopsis* and soybean (*Glycine max*, cv “DN50”) lines overexpressing *GmMYBLJ*, its full-length Coding Sequence (CDS) sequence was amplified and cloned into the YFP-infused vectors, pEarleyGate101 (pEarleyGate101-*GmMYBLJ*) and pTF101 (pTF101-*GmMYBLJ*), respectively. Transgenic *Arabidopsis* and soybean lines were produced using *Agrobacterium*-mediated floral dip (Tsuda et al., 2012) and cotyledonary-node explants (Zeng et al., 2004), respectively. Positive transgenic lines were screened on Murashige and Skoog (MS) medium with 100 mg/L of cephalosporin and 10 mg/L of glufosinate, and confirmed by RT-qPCR using gene-specific primers. Primer information is listed in **Supplementary Table S1**.

To generate *GmMYBLJ*-silenced hairy roots, specific fragments of *GmMYBLJ* were inserted into the RNAi vector pK7GWIGW2D(II). Meanwhile, the CDS sequence of *GmMYBLJ* was cloned into the overexpression vector pK7GM with a Green Fluorescent Protein (*GFP*) fusion, driven by an independent 35S promoter, to generate *GmMYBLJ*-overexpressed hairy roots. The pK7GM-*GmMYBLJ*- and *GmMYBLJ*-silenced vectors were transformed into the *Agrobacterium rhizogenes* strain K599. Transgenic hairy roots were generated following the method described by Li et al. (2012). The transgenic soybean hairy roots were cultured under a 16-h/8-h (light/dark) photoperiod cycle. After 6 weeks, positive hairy roots were identified by green fluorescence using LUYOR-3415CV (hand lighting) and PCR analysis.

### 2.3 Generation of transgenic GUS reporter lines and GUS staining analysis

To generate transgenic β-glucuronidase (GUS) reporter lines, the promoter region spanning 1,500 bp upstream of the translational start site was cloned and constructed into the Gateway vector pMDC162 to obtain *pGmMYBJ-GUS*. Transgenic *Arabidopsis* plants were generated using the *Agrobacterium*-mediated floral dip method (Tsuda et al., 2012). Positive transgenic lines were screened on MS with 200 mg/L of cephalosporin and 50 mg/L of hygromycin, followed by RT-PCR analysis for confirmation. Primer information is listed in **Supplementary Table S1**.

Histochemical GUS staining was carried out using different tissues from transgenic samples, including 7-day-old seedlings, roots, stems, cauline leaves, inflorescences, silique, rosette leaves at different positions (first to ninth), and leaves under dark condition for 5 days. After vacuum infiltration for 15 min, the samples were incubated in staining solution at 37°C overnight. The tissues were decolorized with 70% ethanol to completely remove chlorophylls, and photographs were taken with a stereomicroscope.

### 2.4 Treatment of soybean for the analysis of circadian rhythm

Soybean seeds were planted and grown under a 12-h light/12-h dark cycle at 22°C for 10 days, after which they were subsequently transferred to constant light conditions. Soybean samples were harvested every 4 h from 0 h to 72 h.

### 2.5 DAB staining analysis

Samples were collected for DAB staining analysis, including 30-day-old *Arabidopsis* plants overexpressing *GmMYBLJ* and wild-type plants, as well as *GmMYBLJ*-overexpressed and *GmMYBLJ*-silenced hairy roots. DAB staining was conducted according to the method described by Daudi and O’Brien (2012). Three biological repeats with three technical repeats were performed.

### 2.6 Stress treatments and measurement of MDA and chlorophyll contents

Two-week-old soybean seedlings were subjected to the following treatments as described previously (Bian et al., 2017): (1) Seedlings were grown under dark conditions, and samples were collected at 0, 1, 2, 3, 4, 5, and 6 days after dark treatment; (2) Salicylic Acid (SA) (2 mM) and ABA (100 μM) were sprayed on the leaves of seedlings, respectively, and samples were collected at 0, 3, 6, 12, 24, 48, and 72 h after SA and ABA treatments; (3) Cold treatment (4°C) and 200 mM NaCl were individually applied to the seedlings, and samples were collected at 0, 3, 6, 12, 24, 48, and 72 h after the salt and cold treatments; and (4) Water supply was withheld for drought stress, and samples were collected at 0, 2, 4, 6, 8, and 10 days of water restriction.

To investigate the effects of *GmMYBLJ* on dark- and stress-induced senescence, 25-day-old transgenic *Arabidopsis* plants overexpressing *GmMYBLJ* and wild-type plants were subjected to dark treatment and *Pst* DC3000 infection for 5 days. Samples were then harvested to measure the contents of malondialdehyde (MDA) and chlorophylls.

To investigate the effects of *GmMYBLJ* on salt-induced senescence, 200 mM NaCl was applied to 10-day-old transgenic soybean plants and nontransformed control for 10 days. Samples were then harvested to measure the MDA content.

The contents of MDA and chlorophylls were determined using the methods described previously (Li et al., 2020; Zhang et al., 2021). Three biological repeats with three technical repeats were performed for each sample. One-way analysis of variance (ANOVA) with Least Significant Difference (LSD) test was used to calculate the statistical significance, and significant differences are indicated by an asterisk (^*^ *p* < 0.05; ^**^ *p* < 0.01; ^***^ *p* < 0.001).

### 2.7 RT-qPCR analysis

Total RNA from *Arabidopsis* and soybean samples were extracted using the RNAprep Pure Plant Plus Kit (Tiangen, Beijing, China). First-strand cDNA was generated using StarScript II RT Mix with gDNA Remover (GenStar, Beijing, China), following the manufacturer’s instructions. RT-qPCR was then performed using an ABI stepOnePlus PCR system and SYBR Premix Ex Taq (Takara, Beijing, China), with *GmSUBI3* and *AtACTIN8* used as internal controls for soybean and *Arabidopsis* samples, respectively. Three biological replicates with three technique repeats were performed for each sample, and data analysis was conducted using ABI StepOne Plus v2.3 software. One-way ANOVA with LSD test was used to calculate the statistical significance, with significant difference indicated by an asterisk (^*^ *p* < 0.05; ^**^ *p* < 0.01; ^***^ *p* < 0.001). The primer sequences are listed in **Supplementary Table S1**.

### 2.8 Subcellular localization of *GmMYBLJ*

The open reading frame (ORF) of *GmMYBJ* from *Williams 82* was amplified and subsequently cloned into the pCAMBIA1300 vector, with *YFP* driven by the 35S promoter. The resulting plasmid, pCAMBIA1300-*GmMYBJ-YFP*, and the empty vector, pCAMBIAI1300-*YFP*, were transformed into *Agrobacterium tumefaciens* strain GV3101, and then introduced into the leaves of *Nicotiana benthamiana* (Sparkes et al., 2006). GFP fluorescence in the leaves was observed using a Leica confocal microscope (http://www.leica.com/).

### 2.9 Phylogenetic and conserved motif analysis

The sequences of GmMYBLJ and its homologous proteins were downloaded from the Phytozome website (https://phytozome-next.jgi.doe.gov). Conserved sequences were predicted on the MEME website (http://meme-suite.org/), and the conserved motifs were visualized using the TBtools software (Chen et al., 2023). Conserved domains were predicted using the tool CDD (https://www.ncbi.nlm.nih.gov/cdd). The phylogenetic tree was constructed by the Neighbor-joining method using MEGA7 (Poisson mode, complete deletion, and 1,000 bootstrap values) (Kumar et al., 2016) and visualized using the EVOLVIEW tool (https://www.evolgenius.info/evolview). The online program STRING was used to predict the interactors of GmMYBLJ (https://string-db.org/).

### 2.10 Yeast one-hybrid assay

To examine the binding of GmMYBLJ to its targets, the full-length CDS of *GmMYBLJ* was cloned and introduced into the vector pB42AD. The binding sites in the promoter region of the three *WRKY* genes (*AtWRKY30*, *AtWRKY45*, *AtWRKY75*) were predicted using the online program PlantCare (https://bioinformatics.psb.ugent.be/webtools/plantcare/html/). The promoter fragments spanning the predicted binding sites were cloned as preys and constructed into the vector pLacZi (*pWRKY30*, 342 bp; *pWRKY45-1*, 372 bp; *pWRKY45-2*, 320 bp; *pWRKY75-1*, 411 bp; *pWRKY75-2*, 469 bp). Negative controls, i.e., pB42AD/pLacZi, pB42AD-*GmMYBLJ*/pLacZi, and pB42AD/pLacZi-*pAtWRKY30*, pB42AD/pLacZi-*pAtWRKY45*, pB42AD/pLacZi-*pAtWRKY75*, and one positive control, pB42AD-*AtRVE8*/placZi-*AtPRR5*, were also included. The primers are listed in **Supplementary Table S1**.

## 3 Results

### 3.1 Identification of *GmMYBLJ* and its subcellular localization

**Figure 1:**
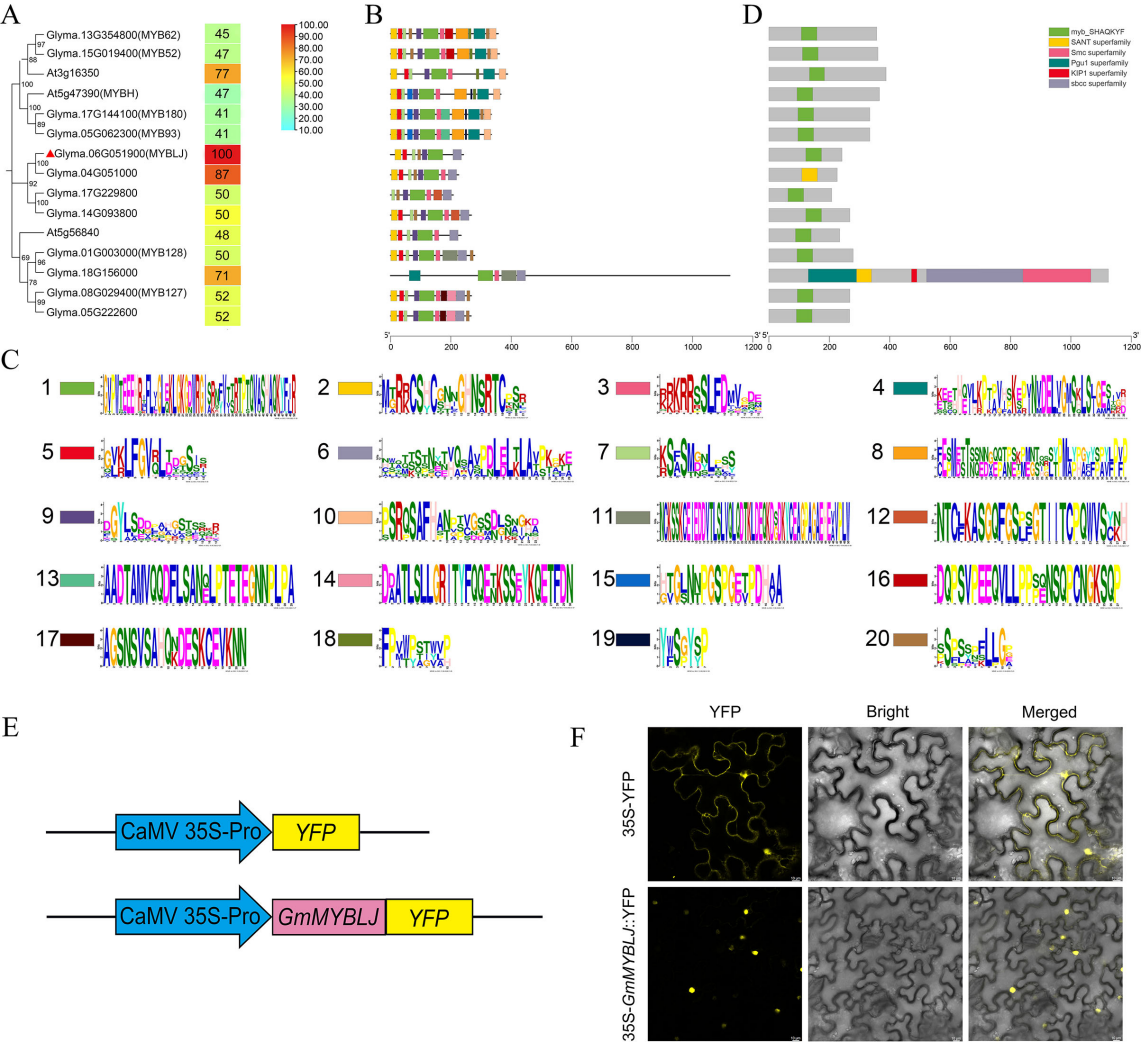
Characterization of GmMYBLJ and its homologous proteins in soybean and *Arabidopsis*. **(A)** Phylogenetic analysis of GmMYBLJ and its homologs in *Arabidopsis* (three AtMYBs) and soybean (11 GmMYBs). GmMYBLJ is marked with a red triangle. All the sequences of MYB proteins were downloaded from the phytozome database (https://phytozome.jgi.doe.gov/pz/portal.html). The protein identities of GmMYBLJ with other MYBs are listed. **(B)** Motif compositions of GmMYBLJ and its homologs. The online program MEME was used to predict motifs, with the maximum number set at 20. **(C)** Sequence logos of the 20 motifs. The height of the letters within each stack represents their relative frequency. Colored symbols correspond to the colored boxes representing motifs. **(D)** Diagrams of conserved domains for the 12 GmMYBs and three AtMYBs. The length of each protein sequence is represented by a gray bar, with colored boxes indicating conserved domains. The sequence length of each protein is shown by a gray line at the bottom. **(E)** Schematics of the two constructed vectors: *p35S-GmMYBJ::YFP* and *p35S-YFP*. **(F)** Subcellular localization of GmMYBLJ. *A*. *tumefaciens* carrying the plasmid *p35S-GmMYBJ::YFP* or *p35S-YFP* was infiltrated into *N. benthamiana* leaves and visualized using confocal microscopy. The scale bar is shown as 10 μm.

To investigate the CCA1-like MYBs associated with leaf senescence, a homology search was performed using the sequence of *Arabidopsis* MYB HYPOCOTYL ELONGATION-RELATED (MYBH) (a leaf senescence-associated regulator) against the 54 CCA1-like MYBs identified previously in soybean (Bian et al., 2017). As a result, 12 CCA1-like MYBs showed high protein identity with AtMYBH and were clustered into three clades (**Figure 1A**). It was further observed that the CCA1-like MYBs within the same clade exhibited similar motif compositions (**Figures 1B, C**). All the proteins contained a MYB domain with the conserved motif SHAQK(Y/F)F (**Figures 1B–D**), implying functional redundancy among these proteins. Subsequently, GmMYBLJ was selected as a representative to investigate the functional roles of soybean CCA1-like MYBs in leaf senescence. The protein identities of the abovementioned 12 MYBs with GmMYBLJ were calculated, ranging from 41% to 87%, with Glyma.04G051000 showing the maximum value (**Figure 1A**). GmMYBLJ is closely related to AtMYBH, with 47% protein identity, suggesting that GmMYBLJ might be involved in the regulation of leaf senescence.

To investigate its subcellular localization, the full ORF of *GmMYBLJ* was fused with yellow fluorescent protein (YFP) to create *p35S-GmMYBLJ::YFP*, with *p35S-YFP* used as a negative control (**Figure 1E**). As shown in **Figure 1F**, YFP fluorescence predominantly appeared in the nucleus of tobacco cells expressing *p35S-GmMYBLJ::YFP*. In comparison, the *p35S-YFP* control exhibited YFP signals in both the nucleus and cytoplasm (**Figure 1F**). These results suggested that *GmMYBLJ* may act as a transcription factor, performing its functions in the nucleus.

### 3.2 Spatiotemporal expression of *GmMYBLJ* and its response to leaf senescence

**Figure 2:**
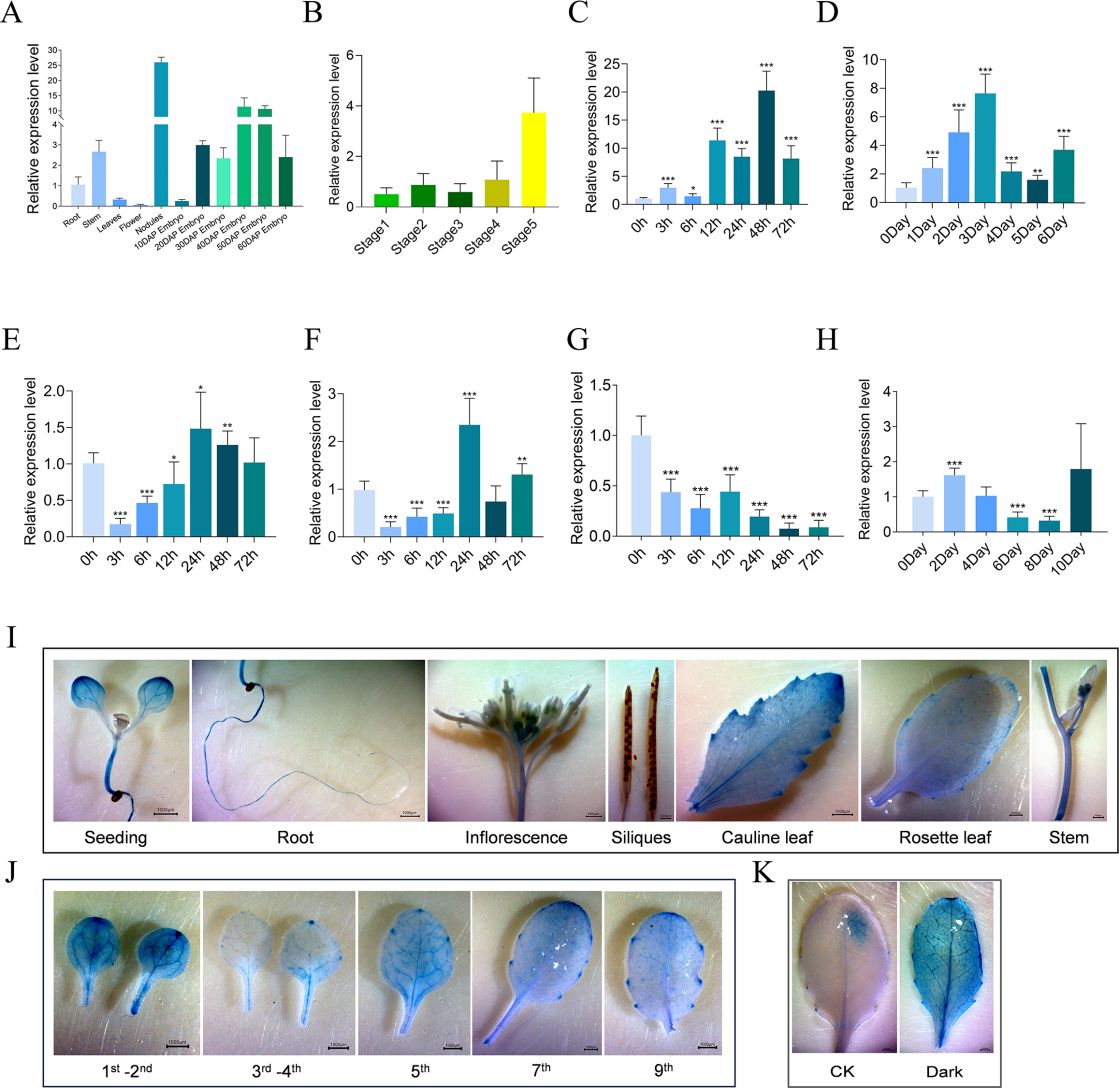
Spatiotemporal expression of *GmMYBLJ* and its response to leaf senescence. **(A, B)** Gene expression of *GmMYBLJ* in various tissues **(A)** and leaves at five developmental stages **(B)**. Tissues include root, stem, leaf, flower, nodule, and embryo at different developmental stages (10, 20, 30, 40, 50, and 60 days after pollination [DAP]). The five developmental stages of leaves correspond to 15, 30, 45, 60, and 75 days after the appearance of the third compound leaves. **(C–H)** Expression pattern of *GmMYBLJ* under various treatments: salt **(C)**, dark **(D)**, SA **(E)**, ABA **(F)**, cold **(G)**, and drought **(H)**. Two-week-old soybean seedlings were subjected to 200 mM NaCl or cold conditions, and samples were collected at 0, 3, 6, 12, 24, 48, and 72 h after treatment. For SA and ABA treatments, 2 mM SA or 100 μM ABA was sprayed on the leaves of 2-week-old soybean seedlings, and samples were similarly collected at 0, 3, 6, 12, 24, 48, and 72 h post-treatment. Two-week-old soybean seedlings were grown under dark conditions, and samples were collected at 0, 1, 2, 3, 4, 5, and 6 days after the dark treatment. For drought stress, water supply was withheld, and samples were collected at 0, 2, 4, 6, 8, and 10 days of water restriction. RT-qPCR analysis was performed with three biological replicates, each comprising three technical replicates. The standard error of the mean is represented by error bars. Data were normalized against the *GmSUBI-3* gene. Statistical significance was calculated using one-way ANOVA with the LSD test, with significant differences indicated by an asterisk (^*^ *p* < 0.05; ^**^ *p* < 0.01; ^***^ *p* < 0.001). **(I–K)** Histochemical analysis of *pGmMYBLJ::GUS* was performed using different various *Arabidopsis* tissues **(I)**, rosette leaves at different positions **(J)**, and under dark conditions **(K)**. Materials for GUS staining include 7-day-old seedlings, roots, stems, cauline leaves, inflorescences, siliques, rosette leaves at different positions (first–ninth), and leaves exposed to dark conditions for 5 days. The scale bar is shown as 1,000 μm.

To provide functional insights into *GmMYBLJ* in soybean, its expression pattern was investigated in roots, stems, leaves, flowers, nodules, and embryos at different developmental stages (10, 20, 30, 40, 50, and 60 DAP). It was found that *GmMYBLJ* exhibited the highest expression in nodules, followed by embryos and stems, with the lowest expression in flowers (**Figure 2A**). Further observation showed that the transcripts of *GmMYBLJ* gradually accumulated as embryos develop (**Figure 2A**), suggesting that *GmMYBLJ* might play important roles during embryo development.

To explore whether *GmMYBLJ* expression changes in responding to leaf senescence, its expression level was examined in soybean leaves at five different stages (15, 30, 45, 60, and 75 days after the appearance of the third compound leaves, **Supplementary Figure S1**). It was shown that the expression of *GmMYBLJ* was relatively low at the first four stages and remarkably increased in the 75-day-old leaf (**Figure 2B**), suggesting that *GmMYBLJ* might play a functional role in the regulation of leaf senescence. Additionally, the expression patterns of *GmMYBLJ* were investigated under the following treatments: darkness (for 0, 1, 2, 3, 4, 5, and 6 days), NaCl (for 0, 3, 6, 12, 24, 48, and 72 h), drought (for 0, 2, 4, 8, and 10 days), cold, SA, and ABA (for 0, 3, 6, 12, 24, 48, and 72 h). The expression of *GmMYBLJ* was significantly increased under NaCl and dark conditions, especially at 3 days after the dark treatment (**Figures 2C, D**), while it was remarkably decreased shortly after the SA and ABA treatments (**Figures 2E, F**), suggesting that phytohormones and darkness might be involved in regulating the expression of *GmMYBLJ*. Additionally, it was observed that the expression of *GmMYBLJ* gradually decreased as the cold treatment progressed (**Figure 2G**), while it decreased at 6 and 8 days after the drought treatment (**Figure 2H**). Unexpectedly, no rhythmicity was observed in the expression of *GmMYBLJ* (**Supplementary Figure S2**).

To test the promoter activity of *GmMYBLJ*, we cloned the promoter region (1,500 bp upstream of the translational start site) and fused it with the *GUS* gene. Subsequently, transgenic *Arabidopsis* plants were generated as reporter lines. Histochemical staining analysis indicated that GUS signals appeared in seedlings, roots, anthers, cauline leaves, rosette leaves, and stems at varied intensities, while no signal was detected in siliques (**Figure 2I**). Furthermore, the promoter activity was investigated in the leaves at different positions (first–ninth) of the rosette using transgenic *Arabidopsis* reporter lines. It was found that cotyledons showed more GUS signals than other rosette leaves (**Figure 2J**), Additionally, it was observed that GUS signals in the leaves were stronger under dark conditions than normal conditions (**Figure 2K**). These results suggest that the promoter of *GmMYBLJ* might exhibit higher activity in senescent leaves or under dark conditions.

### 3.3 *GmMYBLJ* affects plant morphology and confers leaf senescence in *Arabidopsis*

**Figure 3:**
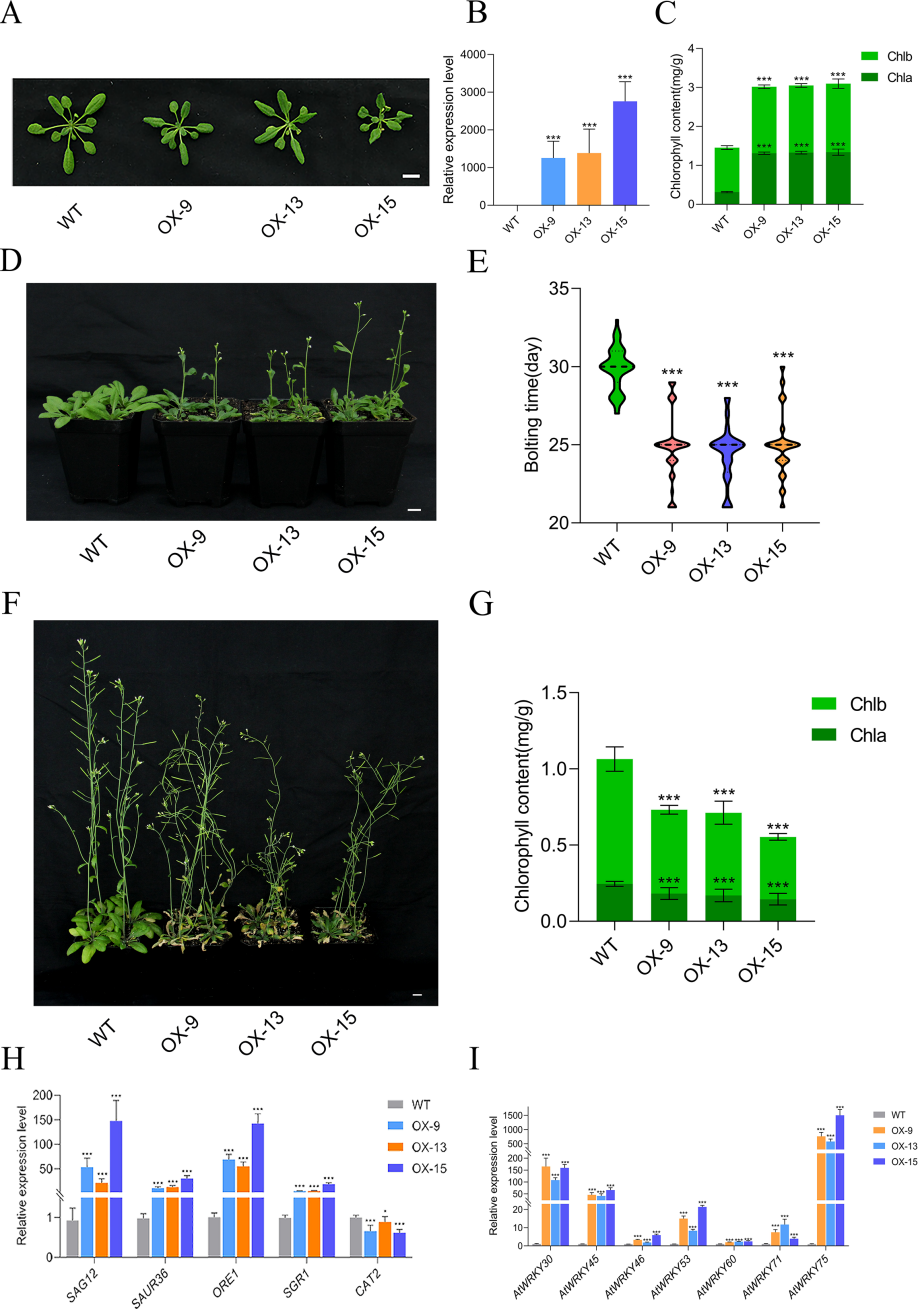
*GmMYBLJ* influences morphology and induces leaf senescence in *Arabidopsis*. **(A)** Photograph of 25-day-old transgenic *Arabidopsis* plants overexpressing *GmMYBLJ* and wild type (WT). Three transgenic lines are designated as OX-9, OX13, and OX15. **(B, C)** Relative expression of *GmMYBLJ* **(B)** and chlorophyll content **(C)** in the three transgenic *Arabidopsis* lines (25-day-old). **(D, E)** Photograph **(D)** and comparison **(E)** of bolting time among the three transgenic *Arabidopsis* lines and WT. **(F, G)** Photograph **(F)** and chlorophyll content **(G)** of 45-day-old transgenic *Arabidopsis* plants overexpressing *GmMYBLJ* and WT. **(H, I)** Expression patterns of senescence-associated genes in the three transgenic *Arabidopsis* lines and WT. The senescence-associated genes include *SENESCENCE-ASSOCIATED GENE 12* (*SAG12*), *SMALL AUXIN UP RNA 36* (*SAUR36*), *ORESARA 1* (*ORE1*), *STAY-GREEN 1* (*SGR1*), and seven *WRKY*s (*AtWRKY53*, *AtWRKY75*, *AtWRKY60*, *AtWRKY71*, *AtWRKY45*, *AtWRKY6*, and *AtWRKY46*). The data were normalized against the *AtACT8* gene for all the RT-qPCR analyses. Three biological replicates and three technical replicates for each biological replicate were carried out for all the analyses. The standard error of the mean is represented by an error bar. The one-way ANOVA with LSD test was used to calculate the statistical significance, and the significant difference is indicated by an asterisk (^***^ *p* < 0.001). The scale bar is shown as 1 cm.

To investigate the functional roles of *GmMYBLJ*, transgenic *Arabidopsis* lines overexpressing *GmMYBLJ* were generated and confirmed using RT-qPCR (**Figures 3A, B**). Three transgenic *Arabidopsis* lines (OX-9, OX-13, OX-15) exhibited multiple morphological phenotypes (such as smaller size, darker and curly leaves) as compared to wild type (WT) (**Figures 3A, B**). Further investigation revealed that the total chlorophyll content in the three transgenic lines (25-day-old) reached 3 mg/g, which was significantly higher than that in WT plants (**Figure 3C**). Additionally, transgenic plants exhibited an early flowering phenotype, with the average bolting time of 25 days, occurring 5 days earlier than in WT plants, which bolted at 30 days (**Figures 3D, E**).

Notably, overexpression of *GmMYBLJ* led to leaf senescence in 45-day-old plants, as evidenced by the dried and dead rosette leaves (**Figure 3F**). Chlorophyll content was then measured in the fourth and fifth rosette leaves, revealing a 31.27%–47.98% reduction in total chlorophyll content in transgenic *Arabidopsis* leaves as compared to WT (**Figure 3G**). This suggests that *GmMYBLJ* possibly promoted leaf senescence in *Arabidopsis*. Furthermore, the expression patterns of leaf senescence-associated genes, including *SENESCENCE-ASSOCIATED GENE 12* (*SAG12*), *SMALL AUXIN UP RNA 36* (*SAUR36*), *ORESARA 1* (*ORE1*), *STAY-GREEN 1* (*SGR1*), and *CATALASE2* (*CAT2*), were investigated in the three transgenic *Arabidopsis* lines, as demonstrated previously (Lei et al., 2023). It was found that the expression of *AtSAG12*, *AtSAUR36*, *AtORE1*, and *AtSGR1* was significantly upregulated by *GmMYBLJ* overexpression, particularly *AtSAG12* and *AtORE1*, which showed 23.1–159.6-fold changes (**Figure 3H**). In contrast, *AtCAT2* was transcriptionally downregulated by *GmMYBLJ* overexpression (**Figure 3H**). In addition, seven *WRKY* genes were selected to investigate their expression patterns, as they have been reported to play important roles in leaf senescence, including *AtWRKY53*, *AtWRKY75*, *AtWRKY60*, *AtWRKY71*, *AtWRKY45*, *AtWRKY6*, and *AtWRKY46* (Cao et al., 2023). As shown in **Figure 3I**, all seven *WRKYs* were transcriptionally elevated in the three transgenic *Arabidopsis* lines overexpressing *GmMYBLJ*. In particular, *AtWRKY30*, *AtWRKY45*, and *AtWRKY75* were upregulated by 40–1,500-fold (**Figure 3I**). Subsequently, the three *WRKY* genes were subjected to yeast one-hybrid (Y1H) analysis to investigate whether they could be bound by GmMYBLJ. No blue colonies were observed when GmMYBLJ was used as the bait and the promoter fragments of the three *WRKY* genes as prey (**Supplementary Figure S3**).

To investigate whether GmMYBLJ interacts with other transcription factors such as WRKYs and NACs, the online program STRING was used to predict potential interactors of GmMYBLJ. As shown in **Supplementary Figure S4**, no WRKY or NAC proteins were predicted to interact with GmMYBLJ. Instead, the following four GmMYBs were predicted to have the potential to interact with GmMYBLJ: Glyma.04G004100 (MYB047), Glyma.06G003800 (MYB48), Glyma.11G107100 (MYBZ2), and Glyma.12G032200 (MYB54).

### 3.4 *GmMYBLJ* accelerates darkness and *Pst* DC3000 infection-induced leaf senescence in *Arabidopsis*

**Figure 4:**
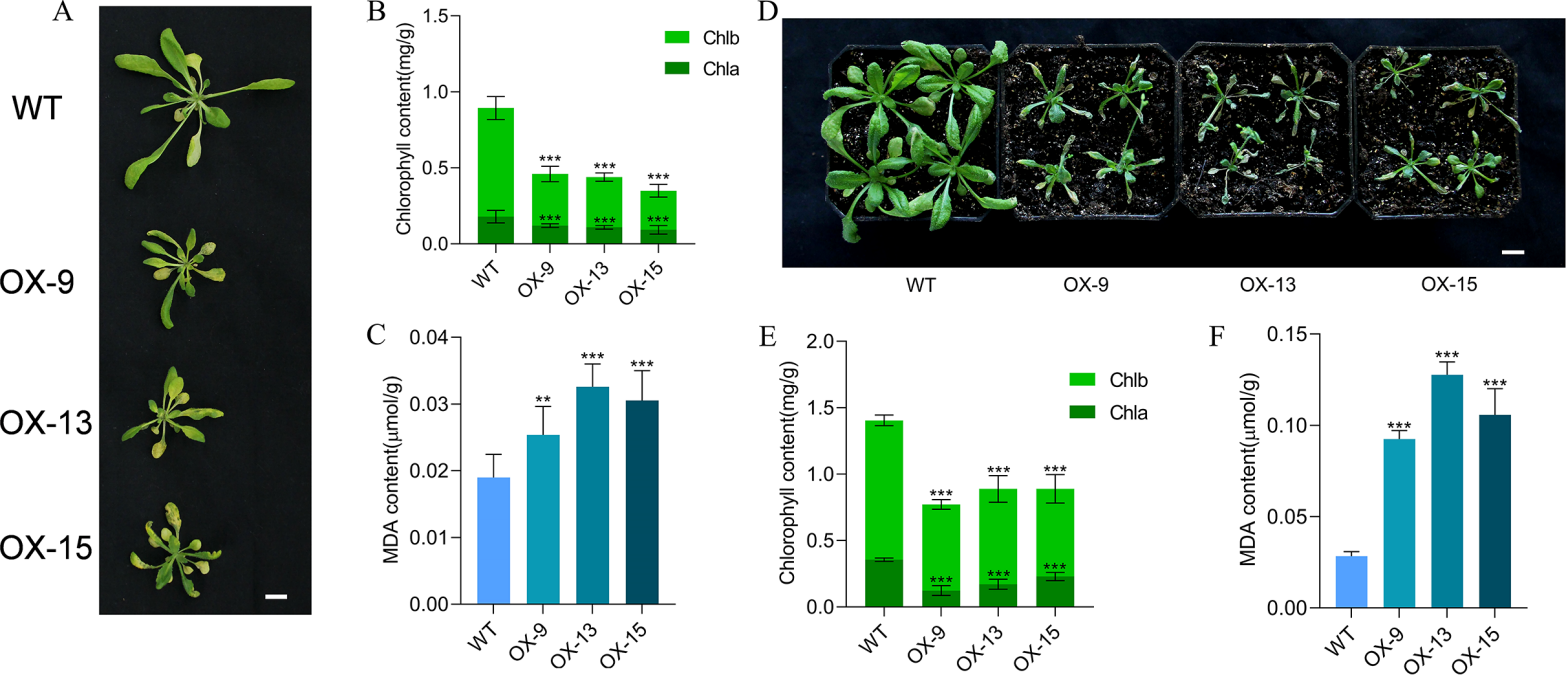
*GmMYBLJ* accelerates dark and *Pst* DC3000-induced leaf senescence in *Arabidopsis*. **(A)** Photograph of 4-week-old transgenic *Arabidopsis* plants overexpressing *GmMYBLJ* and wild type (WT) under dark conditions for 5 days. Three transgenic lines are designated as OX-9, OX13, and OX15. **(B, C)** The contents of chlorophylls **(B)** and MDA **(C)** in the three transgenic lines and WT under dark conditions. **(D)** Photograph of 4-week-old transgenic *Arabidopsis* plants overexpressing *GmMYBLJ* and WT under *Pst* DC3000-treated condition for 5 days. **(E, F)** The contents of chlorophylls **(E)** and MDA **(F)** in the three transgenic lines and WT under *Pst* DC3000 stress. Three biological replicates and three technical replicates for each biological replicate were performed for all the analyses. The standard error of the mean is represented by an error bar. A one-way ANOVA with LSD test was used to calculate statistical significance, and significant differences are indicated by an asterisk (^**^ *p* < 0.01; ^***^ *p* < 0.001). The scale bar is shown as 1 cm.

A large number of studies have demonstrated that darkness promotes leaf senescence (Liebsch and Keech, 2016). To test the role of *GmMYBLJ* in dark-induced leaf senescence, 4-week-old transgenic *Arabidopsis* plants were treated in the dark for 5 days. As shown in **Figure 4A**, the transgenic *Arabidopsis* plants exhibited more severe leaf senescence compared to WT. Further investigation revealed that the chlorophyll content in transgenic *Arabidopsis* leaves was significantly reduced, with a 48.54%–60.86% reduction compared to WT (**Figure 4B**). Conversely, the MDA content increased significantly by 33.68%–71.58% (**Figure 4C**), suggesting that *GmMYBLJ* may accelerate dark-induced leaf senescence in *Arabidopsis*.

Biotic stress is another factor that induces plant leaf senescence (Antonietta et al., 2024). To explore the effect of *GmMYBLJ* on leaf senescence under biotic stress conditions, 4-week-old plants of the three transgenic *Arabidopsis* lines were treated with the bacterial pathogen *Pst* DC3000 for 5 days. As shown in **Figure 4D**, the transgenic *Arabidopsis* plants exhibited more severe leaf senescence compared to the WT. Further investigation revealed that the chlorophyll content in the transgenic *Arabidopsis* plants was significantly lower compared to the WT (**Figure 4E**), while the MDA content increased by 2.25–3.49-fold (**Figure 4F**), suggesting that *GmMYBLJ* overexpression accelerated *Pst* DC3000-induced leaf senescence in *Arabidopsis*.

### 3.5 *GmMYBLJ* causes ROS accumulation in *Arabidopsis* and soybean hairy roots

**Figure 5:**
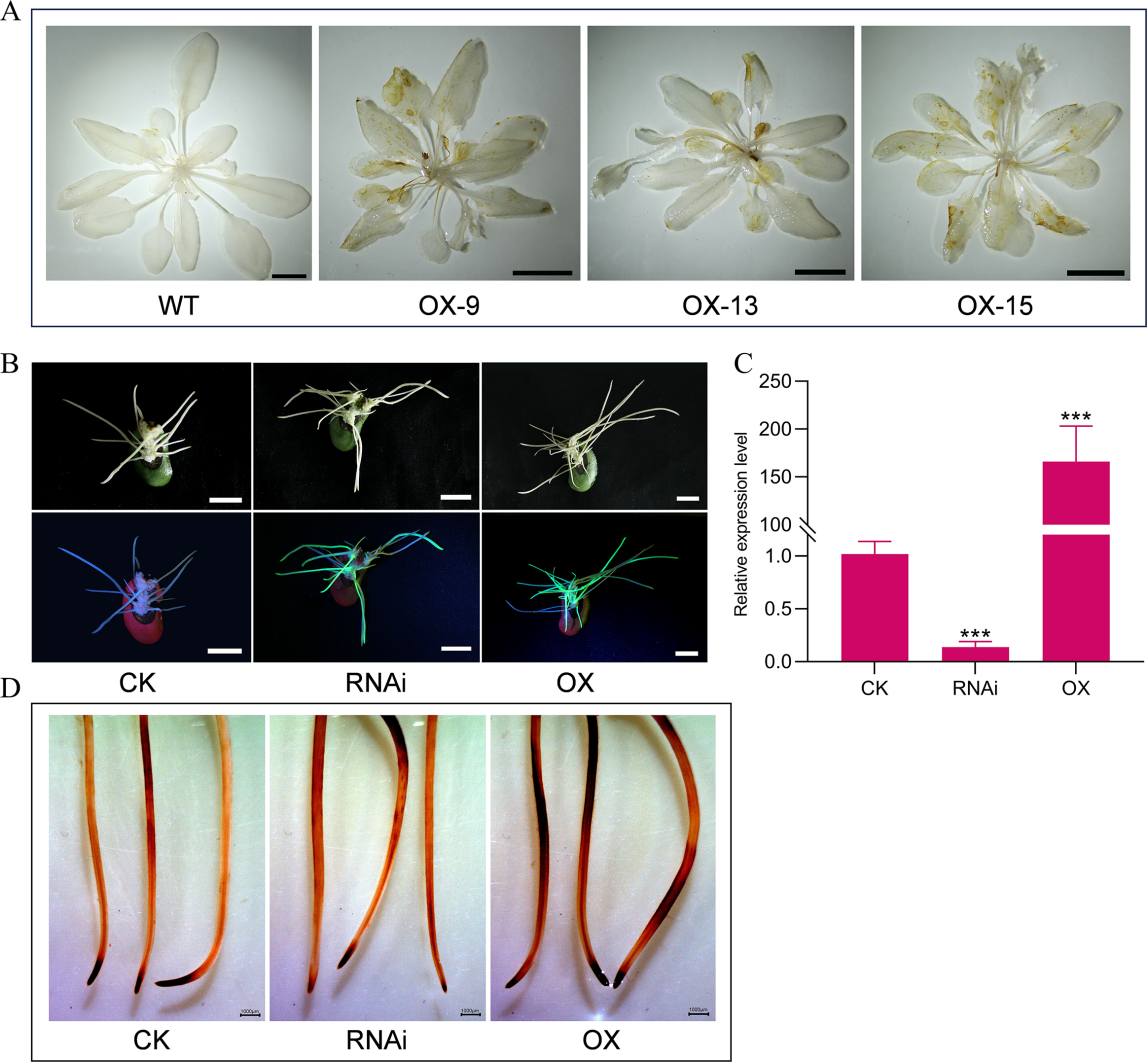
*GmMYBLJ* causes ROS accumulation in *Arabidopsis* and soybean hairy roots. **(A)** DAB staining analysis of 30-day-old *Arabidopsis* plants from the three transgenic lines overexpressing *GmMYBLJ* and wild type (WT). The scale bar is shown as 1 cm. **(B)** Photographs of soybean hairy roots overexpressing and silencing *GmMYBLJ*. The scale bar is shown as 1 cm. **(C)** Expression level of *GmMYBLJ* in soybean hairy roots overexpressing and silencing *GmMYBLJ*. **(D)** DAB staining analysis of soybean hairy roots overexpressing and silencing *GmMYBLJ*. The data were normalized against *GmSUBI-3* for RT-qPCR analysis. The standard error of the mean is represented by an error bar. The one-way ANOVA with LSD test was used to calculate the statistical significance, and the significant difference is indicated by an asterisk (^***^ *p* < 0.001). Three biological replicates and three technical replicates for each biological replicate were performed for all the analyses. The scale bar is shown as 1,000 μm.

Reactive oxygen species (ROS) play a crucial role in the process of plant organ senescence (Asad et al., 2024). To investigate whether *GmMYBLJ* is involved in regulating leaf senescence through the ROS-mediated pathway, DAB staining analysis was performed using 30-day-old transgenic *Arabidopsis* plants overexpressing *GmMYBLJ*. As shown in **Figure 5A**, a brown color clearly appeared on the leaves of the transgenic *Arabidopsis* plants, indicating that overexpression of *GmMYBLJ* likely resulted in increased ROS production. Furthermore, *GmMYBLJ*-overexpressed and *GmMYBLJ*-silenced soybean hairy roots were individually generated using *A. rhizogenes*-mediated hairy root transformation. Positive transgenic hairy roots were identified by LUYOR-3415CV handheld lamp irradiation and RT-qPCR (**Figures 5B, C**). The transgenic roots were then collected for DAB staining analysis. As shown in **Figure 5D**, *GmMYBLJ*-overexpressed hairy roots showed more brown color after DAB staining, while *GmMYBLJ*-silenced hairy roots exhibited less coloration (**Figure 5D**). These results suggest that *GmMYBLJ* may positively regulate the accumulation of ROS in soybean hairy roots.

### 3.6 *GmMYBLJ* overexpression accelerates salt-induced senescence in soybean

**Figure 6:**
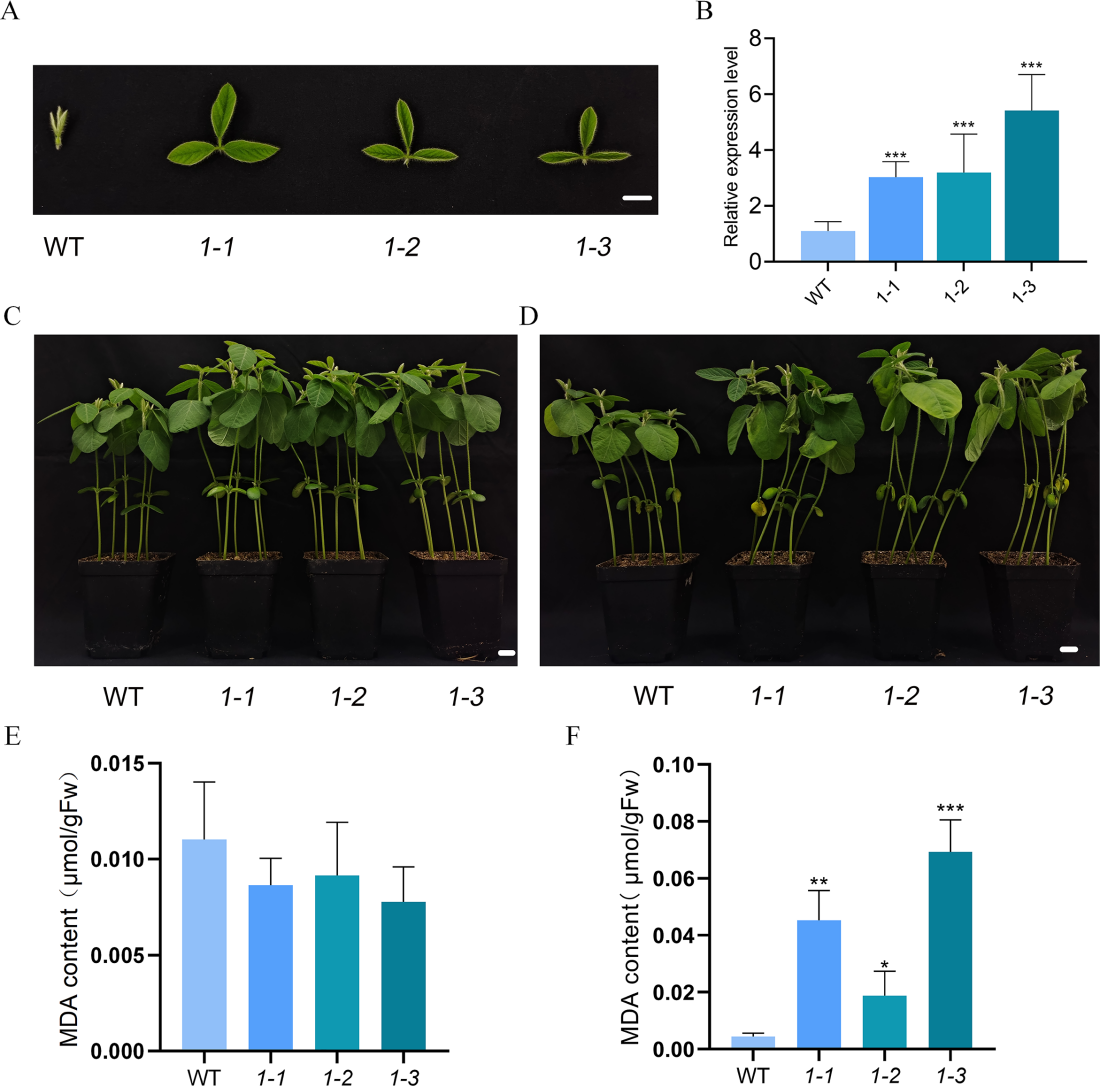
GmMYBLJ accelerates salt-induced senescence in soybean. **(A)** Photographs of the second round of the three compound leaves of 18-day-old transgenic soybean plants overexpressing *GmMYBLJ*. **(B)** The relative expression level of *GmMYBLJ* in the transgenic soybean plants. **(C, D)** Photographs of 10-day-old transgenic soybean plants and untransformed control (WT) grown under normal conditions **(C)** and treated with 200 mM NaCl **(D)** for 10 days. **(E, F)** MDA content of 10-day-old transgenic soybean plants and WT grown under normal conditions **(E)** and treated with 200 mM NaCl **(F)** for 10 days. The standard error of the mean is represented by an error bar. One-way ANOVA with LSD test was used to calculate statistical significance, and significant differences are indicated by an asterisk (^*^ *p* < 0.05; ^**^ *p* < 0.01; ^***^ *p* < 0.001). Three biological replicates and three technical replicates for each biological replicate were conducted for all the analyses. The scale bar is shown as 1 cm.

To investigate the effects of *GmMYBLJ* on leaf senescence in soybean, transgenic soybean plants overexpressing *GmMYBLJ* were generated and verified using RT-qPCR (**Figures 6A, B**). Ten-day-old transgenic soybean plants were subjected to salt stress for 10 days. Under normal conditions, the transgenic plants exhibited faster growth and greater height compared to WT (**Figure 6C**), with no significant difference detected in MDA levels (**Figure 6D**). When subjected to salt stress, transgenic soybean plants exhibited more severe leaf wilting compared to WT (**Figure 6E**), and the MDA content increased by 3.21–14.56-fold relative to WT (**Figure 6F**), suggesting that the overexpression of *GmMYBLJ* accelerates salt-induced senescence in soybean.

## 4 Discussion

MYB transcription factors have been considered important players in the regulation of leaf senescence. To date, genome sequence availability has allowed the identification of a few hundred *MYBs* in soybean (Du et al., 2012). However, little is known about their functional roles in the progression of leaf senescence. In this study, a soybean *CCA1*-*like* gene, *GmMYBLJ*, was identified as playing a role in the regulation of leaf senescence, laying the foundation for further investigation into its regulatory mechanisms.

MYBs constitute one of the most important transcription factor families in plants, performing functions in virtually every aspect of plant growth and development, as well as response to stresses (Thakur and Vasudev, 2022; Biswas et al., 2023). In many plant species, MYBs have been shown to positively regulate leaf senescence. For example, overexpression of *AtMYBL*, *AtMYBH*, and *NtMYB1* accelerated leaf senescence (Zhang et al., 2011; Huang et al., 2015; Yang et al., 2023). However, it has also been reported that *AtMYB59* and *AtGLK*s act as negative regulators of leaf senescence (Zhang et al., 2022; He et al., 2023). In this study, we provide evidence supporting that soybean *MYBLJ* might be a positive regulator in the process of leaf senescence. The most direct evidence is that overexpression of *GmMYBLJ* in *Arabidopsis* directly accelerated leaf senescence (**Figures 3F, G**). Meanwhile, *GmMYBLJ* itself exhibited an age-dependent expression pattern and promoter activity (**Figures 2B, J**). Moreover, *AtSAG12* is considered one of the most important senescence-associated marker genes for characterizing leaf senescence (Gombert et al., 2006). Our results showed that the expression of *AtSAG12* was significantly elevated by the overexpression of *GmMYBLJ* (**Figure 3G**). Notably, GmMYBLJ is closely related to AtMYBH, with 47% protein identity (**Figure 1A**). *AtMYBH* has been reported to play positive roles in both developmentally regulated and dark-induced leaf senescence (Huang et al., 2015). Therefore, we proposed that *GmMYBLJ* might be positively involved in the regulation of leaf senescence, similar to its homologous gene *AtMYBH*. Additionally, the high protein identity of GmMYBLJ with other GmMYBs, such as Glyma.04G051000 (as shown in **Figure 1A**), suggests that there may be other *GmMYB*(s) that complement the functions of *GmMYBLJ* in the regulation of soybean senescence.

It is well known that biotic and abiotic stresses can accelerate leaf senescence and programmed cell death in most crop plants, leading to the cessation of photosynthesis, transpiration, and other assimilatory processes (Fraga et al., 2021; Lei et al., 2023). Numerous studies have shown that delaying senescence can enhance plant resistance to stress (Zhou and Yang, 2023; Antonietta et al., 2024). However, in some cases, delayed senescence did not impact stress resistance (Antonietta et al., 2024). In this study, GUS staining and expression pattern analysis showed that *GmMYBLJ* might be transcriptionally induced by dark and salt stresses (**Figures 2C, F, K**). Its overexpression in *Arabidopsis* or soybean accelerated leaf senescence and MDA accumulation under dark conditions, upon *Pst* DC3000 infection, or salt stress (**Figures 4**, **6**), supporting the notion that *GmMYBLJ* not only promotes leaf senescence under normal conditions but also accelerates stress-induced senescence. Generally, a course of action interacts with the ROS burst during aging and senescence progression (Niu et al., 2020; Shirzadian-Khorramabad et al., 2022). In this study, transgenic *Arabidopsis* plants overexpressing *GmMYBLJ* showed greater MDA accumulation compared to WT (**Figures 4C, F**). Moreover, brown coloration clearly appeared on transgenic *Arabidopsis* leaves based on DAB-mediated ROS detection (**Figure 5A**). These results suggest that *GmMYBLJ* may perform its functions through a ROS-associated pathway. Numerous studies have indicated that *NACs* and *WRKYs* play pivotal roles in the regulation of leaf senescence (Ferreira et al., 2020; Niu et al., 2020; Zhang et al., 2022). Consistently, our data showed that *AtORE1/AtNAC2*, *AtWRKY75*, *AtWRKY71*, *AtSGR1*, and *AtSAG12* were all remarkably upregulated in transgenic *Arabidopsis* overexpressing *GmMYBLJ* (**Figures 3H, I**). Previous studies have indicated that *AtORE1/AtNAC2* plays a key role in the onset of leaf senescence by affecting the ROS-mediated pathway and promoting *AtSGR1* expression (Ueda et al., 2020; Doan et al., 2022). Interestingly, some evidence suggests that *AtWRKY71* influences leaf senescence by directly activating *AtORE1/AtNAC2* (Yu et al., 2021), while *AtWRKY75* forms a regulatory loop with SA and ROS to accelerate leaf senescence (Guo et al., 2017). Thus, it is possible that *GmMYBLJ* may play its functional roles during leaf senescence through the *WRKY-ORE1/NAC2*-ROS regulatory module. Additionally, several other *WRKY* family members have been reported to play important roles in leaf senescence, such as *AtWRKY53* (Wang et al., 2023), *AtWRKY45* (Barros et al., 2022), *AtWRKY75* (Zhang et al., 2022), and *AtWRKY30* (Besseau et al., 2012). In this study, all these *WRKYs* were transcriptionally elevated by *GmMYBLJ* overexpression in *Arabidopsis* (**Figure 3I**). It is thus hypothesized that *GmMYBLJ* may promote the expression of leaf senescence-associated genes and ROS production through the *WRKY* genes and *AtORE1/AtNAC2*, thereby accelerating leaf senescence in *Arabidopsis*. Since ROS plays important roles in the regulation of developmental events and responses to stresses as a cellular signaling molecule (Lopez et al., 2024), it will be very interesting to investigate in the future whether a feedback interaction exists between ROS and *GmMYBLJ* in soybean.

Since adverse external factors often accelerate senescence, delayed senescence under stressful conditions is generally considered an indicator of crop resilience (Antonietta et al., 2024). In this study, overexpression of *GmMYBLJ* resulted in increased sensitivity to stresses in both *Arabidopsis* and soybean (**Figures 4**, **6**). Although we did not determine whether *GmMYBLJ* accelerates leaf senescence in soybean, transgenic *Arabidopsis* plants overexpressing *GmMYBLJ* displayed early leaf senescence (**Figure 3**). Seemingly, *GmMYBLJ* may act as an unfavorable gene for crop resistance to stresses. It would be interesting to further investigate whether *GmMYBLJ* can coordinate early leaf senescence with soybean performance under stress.

In conclusion, our findings suggest that *GmMYBLJ* acts as a positive regulator of leaf senescence. Moreover, *GmMYBLJ* accelerated leaf senescence induced by dark, salt, and *Pts* DC3000 stresses. The effect of *GmMYBLJ* on leaf senescence is possibly associated with the *WRKY* family and senescence-associated genes, such as *AtSAG12*, *AtORE1*, and *AtSGR1*. Its regulatory mechanisms require further investigation.

## Data Availability

The datasets presented in this study can be found in online repositories. The names of the repository/repositories and accession number(s) can be found in the article/**Supplementary Material**.

## Supplementary material

Supplementary Figure 1 Photographs of soybean leaves at different developmental stages. Scale bar is shown in 1 cm.

Supplementary Figure 2 The rhythmical expression of *GmMYBLJ* in soybean seedlings.

Supplementary Figure 3 Interaction of GmMYBLJ with the promoters of three *GmWRKY*s using Y1H assay. **(A)** The schematic diagrams of the promoters of three *GmWRKY*s. The blue and yellow downward triangles indicate the elements of MYB binding sites, and the number shows the start site of each element. The light blue bars show the promoter fragments for Y1H assay. **(B)** Interaction assay between GmMYBLJ and the promoters of the three *GmWRKY*s using Y1H assay. pB42AD-AtRVE8/placZi-*AtPRR5* was set as positive control.

Supplementary Figure 4 Predicted interactors of GmMYBLJ using the online program STRING.
