# Supplementary figures and images for: Identification and functional characterization of the MYB transcription factor GmMYBLJ in soybean leaf senescence
Source: Front Plant Sci. 2025 Jan 24;16:1533592. doi: 10.3389/fpls.2025.1533592 (PMC11802812; doi:10.3389/fpls.2025.1533592)

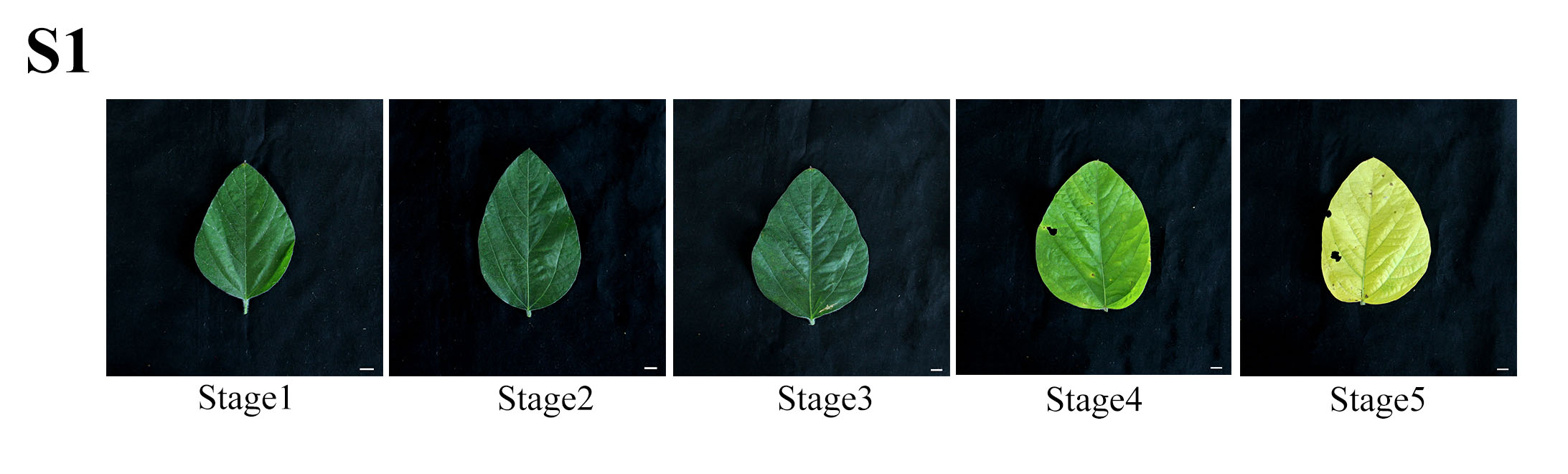

Supplement: Supplementary Figure 1 — Photographs of soybean leaves at different developmental stages. Scale bar is shown in 1 cm. [file Image1.jpeg]

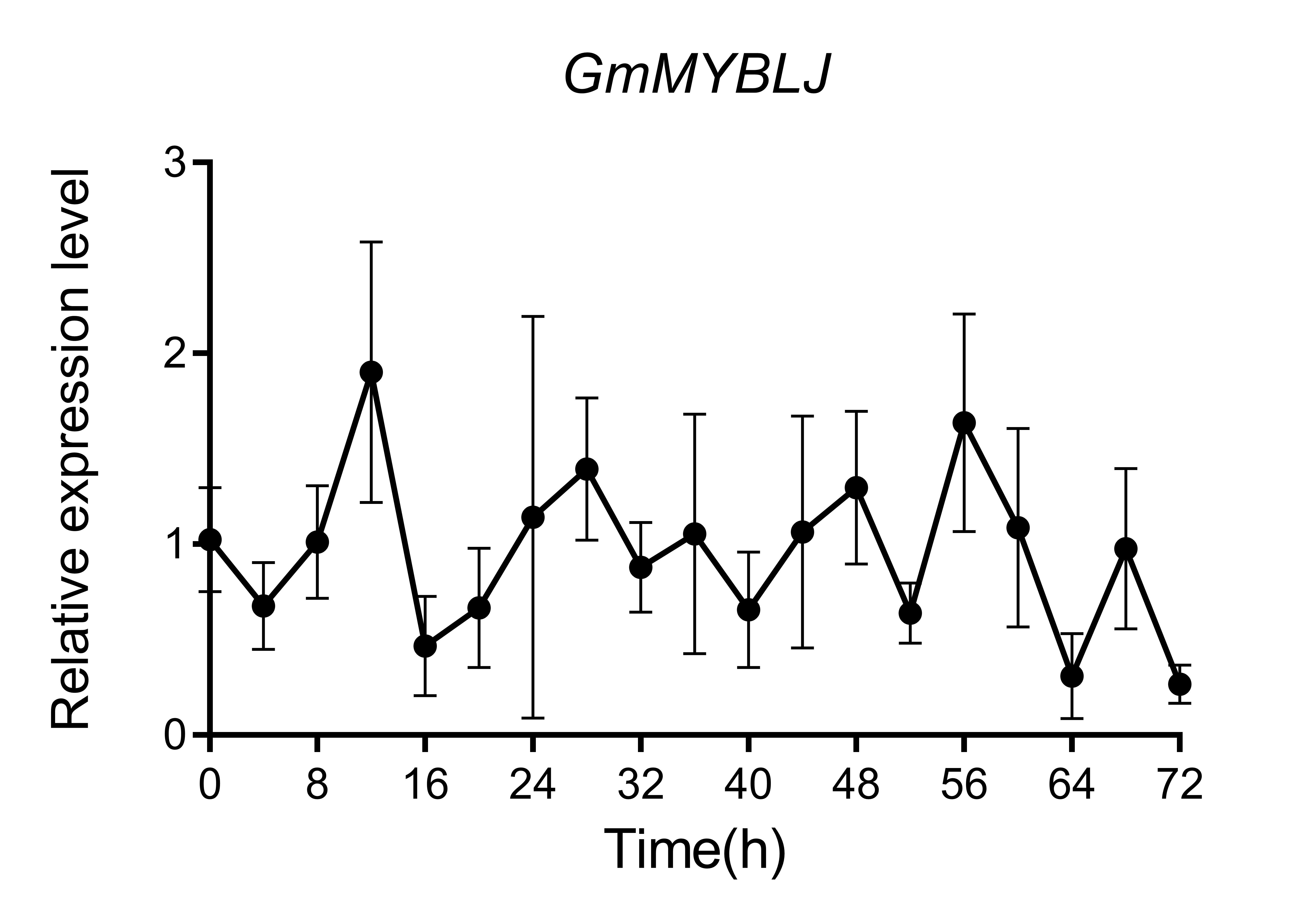

Supplement: Supplementary Figure 2 — The rhythmical expression of GmMYBLJ in soybean seedlings. [file Image2.tif]

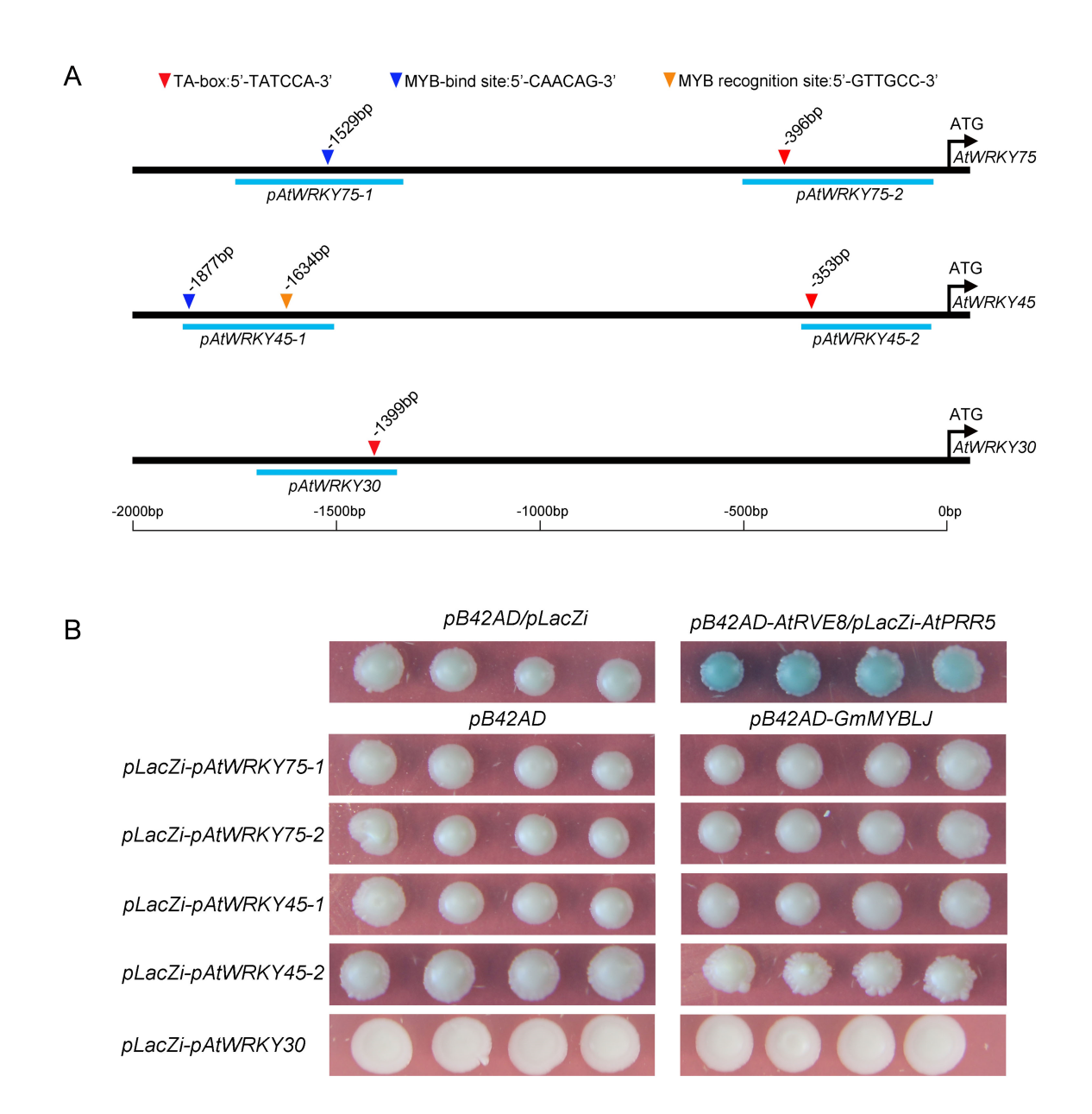

Supplement: Supplementary Figure 3 — Interaction of GmMYBLJ with the promoters of three GmWRKYs using Y1H assay. (A) The schematic diagrams of the promoters of three GmWRKYs. The blue and yellow downward triangles indicate the elements of MYB binding sites, and the number shows the start site of each element. The light blue bars show the promoter fragments for Y1H assay. (B) Interaction assay between GmMYBLJ and the promoters of the three GmWRKYs using Y1H assay. pB42AD-AtRVE8/placZi-AtPRR5 was set as positive control. [file Image3.tif]

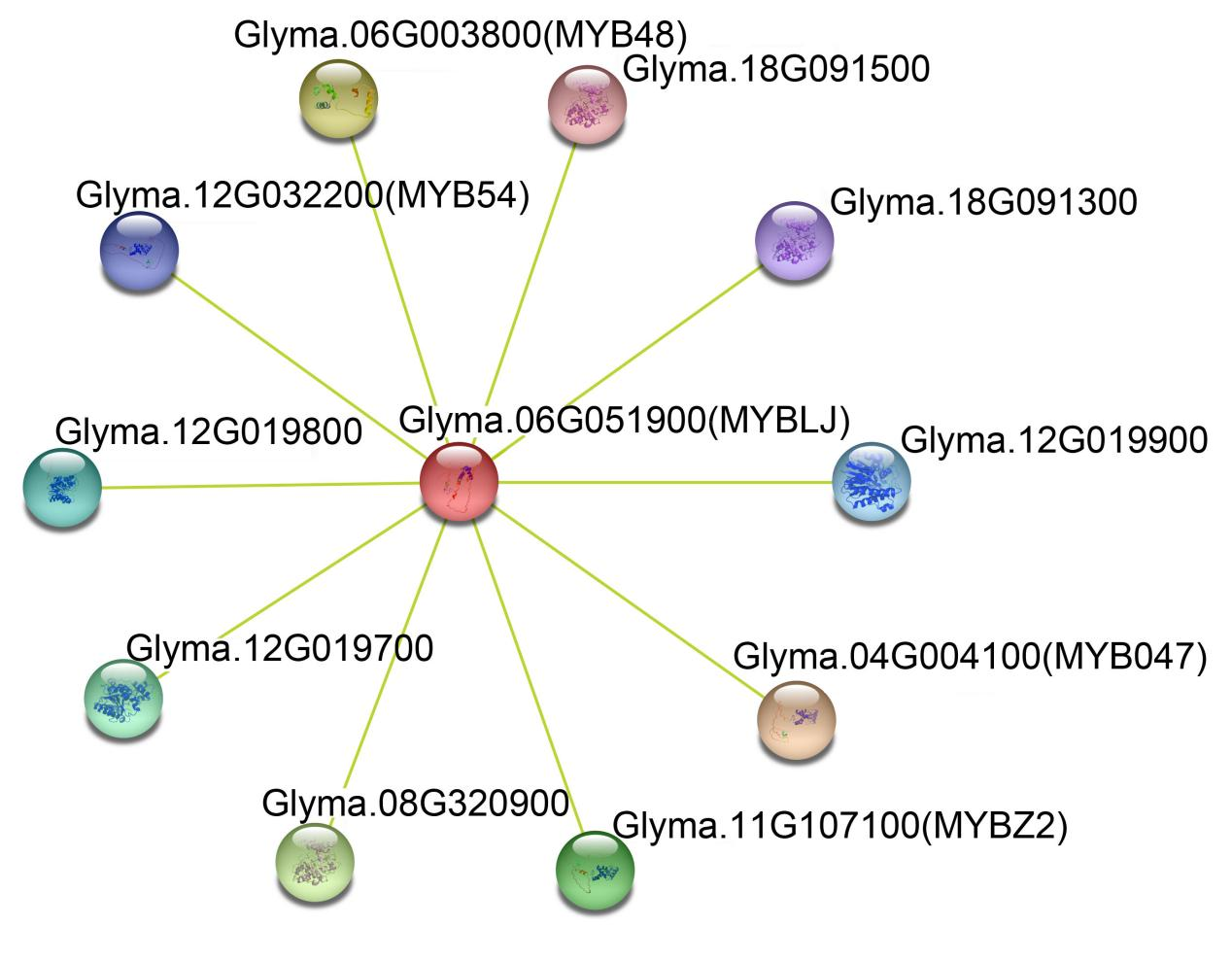

Supplement: Supplementary Figure 4 — Predicted interactors of GmMYBLJ using the online program STRING. [file Image4.tif]
